# Hospital ethical climate survey - selected psychometric properties of the scale and results among polish nurses and midwives

**DOI:** 10.1186/s12912-022-01067-x

**Published:** 2022-11-02

**Authors:** Magdalena Dziurka, Patrycja Ozdoba, Linda Olson, Anna Jedynak, Dorota Ozga, Krzysztof Jurek, Beata Dobrowolska

**Affiliations:** 1 Department of Holistic Care and Nursing Management, Faculty of Health Sciences, Medical University of Lublin, Lublin, Poland; 2 Consultants and previous Professor and Dean at North Park University, Chicago, IL USA; 3 Provincial Polyclinical Hospital, Skierniewice, Poland; 4 Department of Emergency Medicine, Faculty of Medicine, University of Rzeszów, Rzeszów, Poland; 5 Institute of Sociology, Faculty of Social Sciences, John Paul II Catholic University of Lublin, Lublin, Poland

**Keywords:** Hospital ethical climate, Nurses, Midwives, Ethics, Psychometric properties

## Abstract

**Background:**

The hospital ethical climate affects the quality of nursing care. A positive ethical climate is likely to reduce the proportion of those who consider leaving the profession, so it is necessary to develop tools which will enable assessment and analysis of the hospital ethical climate. The aim of this study was to examine selected psychometric properties of the Polish version of the Hospital Ethical Climate Survey, assess the hospital ethical climate perceived by nurses and midwives from Polish hospitals, and to determine its correlations with job-related variables.

**Methods:**

A cross-sectional study among 558 nurses and midwives working in hospitals in Poland.

**Results:**

The 21-item model showed acceptable model fitness between the hypothetical model of ethical climate and the data in the study. Five items with low factor loadings were removed from the study. The internal consistency was satisfactory (0.93). The mean score for the overall hospital ethical climate was 3.62. The highest mean score of hospital ethical climate in the present study was found in the ”peers” subscale and the lowest in the ”physicians” subscale. A positive correlation was found between overall hospital ethical climate and respondents’ satisfaction with work, salary, and working time. The hospital ethical climate was associated with problems found in nurses and midwives’ work, such as: limited time for direct face-to-face care, the lack of equipment and resources to provide high-quality health care, strained relations with hospital managers and other health care professionals, limitations to one’s own competences or those of other medical professionals, moral dilemmas related to patient care, the low prestige of nurses’/midwives‘ work, physical and mental burden, and the risk of making a mistake.

**Conclusion:**

The Polish 21-item version of the Hospital Ethical Climate Survey is a reliable tool. Correlations revealed that relationships with managers and physicians, and working conditions should be improved in order for the hospital ethical climate to improve.

**Supplementary Information:**

The online version contains supplementary material available at 10.1186/s12912-022-01067-x

## Background

Ethical climate can be understood as a combination of employees’ common perceptions in a given organisation, referring to the procedures and policies in place, both standardised and informal, that influence expectations regarding ethical behaviour in the workplace [1]. This view highlights the role of organisations in shaping ethical behaviour among their employees. According to Olson [2–4], an ethical climate centers around work conditions and conduct that trigger discussions and solutions to ethically motivated problems, and is defined as an organisational, manageable and changeable variable which can be used to improve work environment. The organisational conditions and behaviours influence the way in which difficult, ethically-complex patient care issues can be discussed and resolved [5]. The ethical climate of a hospital can impact midwives’ and nurses’ approach regarding ethical decision-making or ethical dilemmas in the workplace. It is influenced by the policies, values and culture of the organisation [6]. The ethical climate plays a meaningful role in terms of behavioural norms, language, rituals, and role models [7] and in the development of moral standards which, for example, hospital employees can recognise and solve [8].

Recently, researchers from around the world have been showing an increased interest in the meaning of ethical climate and in its relation to different variables. For instance, a positive correlation has been observed between the perceptions of ethical climate and nurses’ sense of job satisfaction [9–13], resulting in a higher quality of patient care [14]. At the same time, ethical climate has been found to correlate negatively with one’s intention to change careers [15–17] and with job burnout [9, 18]. A friendly ethical climate is characterised by a shared mission to provide care and by teamwork organised in a manner which allows the team members to exchange experiences, help each other, and jointly contribute to the needs and expectations of patients and their families [19]. An unfriendly ethical climate, on the other hand, aggravates moral stress among nurses [13, 20]. Therefore, with respect to nursing and midwifery work environments, ethical climate constitutes a complex and dynamic factor capable of promoting a healthy atmosphere in a given organisation.

The value of an ethical climate is largely dependent on the relationship between nurses and peers, patients, physicians, management, or the hospital [3]. Cooperation with the physician is based on mutual respect, trust and active participation in decisions about treatment, where managers support the nursing staff in their daily dilemmas and plan effective solutions. Peer relations are based on mutual assistance and the exchange of experiences as well as knowledge and competences. The patient-nurse relationship entails sharing information on patient’s health, providing professional care, and respecting patient’s wishes. As far as the relationship with the hospital is concerned, it mainly involves compliance with hospital guidelines and procedures and, on the other hand, the hospital’s ability to communicate its mission in a clear and concise fashion [3].

Although the importance of the hospital’s ethical climate has been evidenced, including its affiliation with overall care as well as with job satisfaction among health care professionals, this area has not yet been studied in Poland. There is also no tool available in Polish that could be used to conduct research on hospital ethical climate.

## Methods

### Aim

The aim of this study is threefold. First: to examine selected psychometric properties of the Polish version of the Hospital Ethical Climate Survey among nurses and midwives working in hospitals in Poland; second: to assess the hospital ethical climate as perceived by Polish nurses and midwives; and third: to analyse the correlations between the hospital ethical climate and selected job-related variables.

### Study design

A cross-sectional descriptive study was conducted between 2019 and 2020 in the eastern and southern regions of Poland in conformity with the STROBE (Strengthening the Reporting of Observational studies in Epidemiology) guidelines [21].

### Study participants

Study inclusion criteria were as follows: (1) the license to practice as a nurse or midwife, (2) minimum two years of professional experience as a nurse or midwife (to have a good knowledge of the nature of nursing/midwifery work and rely solely on one’s own experience when answering the questions from the study questionnaire), and (3) working in a hospital as a nurse or midwife during conducting the present study. First, the questionnaire was delivered to nurses and midwives (n = 683) who agreed to take part in the study and who work in hospitals in the Lubelskie and Podkarpackie voivodeships in Poland. After three weeks the same respondents were invited to take part in the study and 140 retests were filled and returned correctly.

### Instruments

1. The Hospital Ethical Climate Survey (HECS) developed by Olson in the 1990s in the USA is a 26-item questionnaire assessed by the respondent on a 5-point Likert scale (from 1 – “almost never true” to 5 – “almost always true”). HECS consists of 5 subscales which examine relations between the respondent and his or her peers (four items: 1, 10, 18, 23), patients (four items: 2, 6, 11, 19), managers (six items: 3, 7, 12, 15, 20, 24), hospital (six items: 4, 8, 13, 16, 21, 25), and physicians (six items: 5, 9, 14, 17, 22, 26). The higher the total result, the more positive the ethical climate of the analysed organisation. The Cronbach’s alpha internal consistency and reliability coefficient for the HESC scale was 0.91 [2–4]. Before the research started, approval for the use of the HECS was obtained from the author of the tool. As of date, the Hospital Ethical Climate Survey has been used for research purposes in both public and private hospitals [22], including among nurses and midwives from intensive care wards [23], psychiatric wards [24], and neonatal and pediatric wards [25]. This indicates the usefulness, universality, and reliability of the tool. The scale underwent validation in, among others, Iran [20], Persian [26], Greece [27] and Sweden [28]. The process of translating and adapting Olson’s HECS to Polish conditions was performed with the use of the International Test Commission Guidelines for Translating and Adapting Tests [29]. The Hospital Ethical Climate Survey was translated separately by two Polish bilingual speakers from English into Polish. Next, both translations were synthesised into one and reviewed by the experts – independent judges. They evaluated clarity and comprehensibility of the individual questions and clarity of the instruction accompanying the scale. Then, an experienced native speaker of English retranslated the Polish version of the scale into English. The structure of the scale was left unchanged (item formats, rating scales, scoring categories). At the final stage, the Polish version of the HECS was delivered to a small group of nurses and midwives in the pre-validation test to assess the correctness of the translation and understanding of the questions.
2. Authors’ self-designed questionnaire containing questions on sociodemographic data and professional characteristics of the respondents as well as selected aspects of their work environments such as: limited time for direct face-to-face care, the lack of equipment and resources to provide high-quality health care, strained relations with hospital managers and other health care professionals, limitations to one’s own competences or those of other medical professionals, moral dilemmas related to patient care, the low prestige of nurses’/midwives‘ work, physical and mental burden, and the risk of making a mistake.

### Data collection

Once permission to collect data was secured from the managers of hospitals in the Lubelskie and Podkarpackie voivodeships in Poland, the researchers delivered printed questionnaires to hospital wards and departments with nursing and midwifery personnel. The questionnaires were distributed among those respondents who verbally consented to participate in the study, in line with the inclusion criteria, and were advised about the purpose and course of data collection (stage 1 – test, stage 2 – retest). The questionnaires contained key information about the study as well as researchers’ contact details, in case of additional questions from respondents. The respondents were also informed that they could resign at any stage. Once completed, each questionnaire was to be put in an envelope and returned to the nurse or midwife in charge, and then collected on an agreed date by the researcher.

### Statistical analysis

The statistical analysis was performed with the use of the IBM SPSS Statistics software after the data had been entered into the database created in Microsoft Excel. Descriptive statistics of the collected data were summarised as mean (M), percentages, and standard deviation (SD) values. Also, the Mann–Whitney U test (Z), ANOVA test (F), and t-test (t) were performed.

Confirmatory Factor Analysis (CFA), and Exploratory Factor Analysis (EFA) were performed. CFA is a measure allowing the comparison of data with the theoretical model. Before running the CFA analysis, basic assumptions with respect to sampling adequacy, possible violations of multi-colinearity, and normality of the data were evaluated using the Kaiser-Meyer-Olkin measure of sampling adequacy, the Durbin-Watson test and box plot, and the Chi-square test for Bartlett’s test of sphericity, respectively. The following goodness-of-fit indices were used: the ratio of Chi-square over the degree of freedom (χ^2^/df; < 5 acceptable), the Root Mean Square Error of Approximation (RMSEA; < 0.08 acceptable), the Standardised Root Mean Square Residual (SRMR; < 0.08 good fit), the Comparative Fit Index (CFI; > 0.90 acceptable), the Tucker-Lewis Index (TLI; > 0.90 acceptable), and Bollen’s Incremental Fit Index (IFI; > 0.90 acceptable).

Cronbach’s alpha was applied to establish the internal consistency of factors in the Polish version of the Hospital Ethical Climate Survey (≥ 0.7 acceptable) and to assess the reliability of the tool; a measurement method (test-retest) was also used to determine the test-retest reliability.

### Ethical issues

The research was conducted after obtaining the approval of the Bioethics Committee at the Medical University of Lublin (ref. no.: KE-0254/267/2020). The participants were informed about the anonymity of the study, the voluntary nature of participation, the aim, the process of data collection, and the right to withdraw from the study at any stage. Respondents who gave their voluntary and informed consent were approved for the study.

## Results

### General characteristics of the participants

The first stage of the study was participated in by 558 respondents. The majority of them (n = 537, 96.2%) were women, with the average age of 36 (SD = 10.05). They were mostly living in urban areas (n = 359, 64.3%), with only 199 (35.7%) coming from rural areas. The vast majority of the respondents (n = 297, 53.2%) had the Master’s degree in midwifery or nursing. At the second stage of the study (retest), 140 respondents from the previous phase participated. A detailed description of demographic data is presented in Table 1.

**Table 1:** Characteristics of the participants [n = 558]

Socio-demographic variables		M	SD
**Age**		36.45	10.05
		**n**	**%**
**Gender**	Female	537	96.2
	Male	21	3.8
**Place of residence**	Rural	199	35.7
	Urban	359	64.3
**Marital status**	Single	116	20.8
	Married	366	65.6
	Divorced	22	3.9
	Widow/er	7	1.3
	Informal relationship	47	8.4
**Religious person**	Yes	533	95.5
	No	25	4.5
**Education**	Medical high school	25	4.5
	Medical vocational school	26	4.7
	Bachelor of Midwifery / Nursing	207	37.1
	Master of Midwifery / Nursing	297	53.2
	PhD in Health Sciences / Medical Sciences	3	0.5

M – mean, SD – standard deviation

### Confirmatory factor analysis (CFA)

In order to test the factor structures of the Polish version of the Hospital Ethical Climate Survey with the use of CFA, the assumptions needed to run the factor analysis were first analysed. Accordingly, the Kaiser-Meyer-Olkin (KMO) test to measure sampling adequacy showed the value of 0.937, and Chi-square for Bartlett’s test of sphericity was found to be significant (χ^2^ = 6792.8, df = 325, *p* < 0.001). In addition, the minimum sample size for factor analysis (requirement: > 200) was met.

CFA models were tested to develop a model that would be parsimonious and ensure the best possible fit with the data. The first model was obtained on the basis of the original five-factor questionnaire with 26 items. We tested two models: with uncorrelated error terms and with correlated error terms. The five-factor structure of the HECS-Pol with uncorrelated error terms fitted the data poorly (χ^2^/df = 3.81; CFI = 0.88; IFI = 0.88; TLI = 0.86; SRMR = 0.052; RMSEA = 0.071). The next model tested this five-factor structure with error terms allowed to correlate (χ^2^/df = 3.25; CFI = 0.91; IFI = 0.91; TLI = 0.90; SRMR = 0.049; RMSEA = 0.064). In this case, the model fit improved, although several factor loadings were still well below 0.5. The five items (Q1, Q2, Q4, Q8, Q9) with low factor loadings were removed from the study. The next model (with 21 variables) tested the five-factor structure of the HECS-Pol with error terms allowed to correlate. CFA results revealed acceptable model fitness between the hypothetical model of ethical climate and the study data (χ^2^/df = 3.76; CFI = 0.92; IFI = 0.92; TLI = 0.91; SRMR = 0.047; RMSEA = 0.070) (see Supplementary Material 1). Completely standardized loadings on final factor structure for the factor loadings for the subscales of HECS-Pol were between 0.512 (Q10) to 0.879 (Q12) (Table 2).

**Table 2:** Completely Standardized Loadings on Final Factor Structure for the HECS-Pol

Item*	Factor loadings				
	***Peers***	***Patients***	***Managers***	***Hospital***	***Physicians***
10: My peers help me with difficult patient care issues/problems.	0.512				
18: I work with competent colleagues.	0.652				
23: Safe patient care is given on my unit.	0.590				
6: Nurses have access to the information necessary to solve a patient care issue/problem.		0.605			
11: Nurses use the information necessary to solve a patient care issue/problem.		0.599			
19: Patients’ wishes are respected.		0.574			
3: When I’m unable to decide what’s right or wrong in a patient care situation, my manager helps me.			0.729		
7: My manager supports me in my decisions about patient care.			0.879		
12: My manager listens to me talk about patient care issues/problems.			0.797		
15: My manager is someone I can trust.			0.770		
20: When my peers are unable to decide what’s right or wrong in a particular patient care situation, I have observed that my manager helps them.			0.837		
24: My manager is someone I respect.			0.643		
25: I am able to practice nursing on my unit as I believe it should be practiced.				0.656	
13: The feelings and values of all parties involved in a patient care issue/problem are taken into account when choosing a course of action.				0.580	
16: Conflict is openly dealt with, not avoided.				0.652	
21: There is a sense of questioning, learning, and seeking creative responses to patient care problems.				0.747	
5: Nurses and physicians trust one another.					0.656
14: I participate in treatment decisions for my patients.					0.547
17: Nurses and physicians here respect each other’s’ opinions, even when they disagree about what is best for patients.					0.750
22: Nurses and physicians respect each other.					0.702
26: Nurses are supported and respected in this hospital.					0.714

*Numbers of items are coming from original version of the scale

### Reliability

The Cronbach’s alphas for the five factors or subscales ranged between 0.62 and 0.90. The overall Cronbach’s alpha coefficient was 0.93. The highest Cronbach’s alpha was found in the ”managers” subscale (0.90) and the lowest in the ”peers” subscale. (0.62) (Table 3).

**Table 3:** Reliability of HECS-Pol

	1	2	3	4	5	Overall
**Peers** [1]	1					
**Patients** [2]	0.660^***^	1				
**Managers** [3]	0.521^***^	0.520^***^	1			
**Hospital** [4]	0.605^***^	0.600^***^	0.728^***^	1		
**Physicians** [5]	0.509^***^	0.561^***^	0.591^***^	0.751^***^	1	
**Cronbach’s alpha HECS-POL**	0.62	0.67	0.90	0.76	0.81	0.93
**Cronbach’s alpha HECS** **(Olson, 1998)**	0.73	0.68	0.92	0.77	0.81	0.91
**Test – retest [t - value]**	-0.275	-1.144	1.020	0.416	-1.904	-
**Test – retest [Df - value]**	139	139	139	139	139	-
**Test – retest [p - value]**	0.784	0.151	0.310	0.678	0.059	-

t – t-test, Df – Degrees of freedom, *** <0.001

### Test-retest reliability

A reliability analysis of the test-retest (n = 140), which addressed the consistency of the scores obtained by the same respondents when reexamined with the same test after three weeks, did not reveal any differences between the means from measurement 1 (test) and measurement 2 (retest) (Table 3).

### Construct validity

The interrelation of the ethical climate subscales was investigated. As can be seen in Table 3, the correlations ranged from 0.509 to 0.751. The highest correlation was found between subscales ”physicians” and ”hospital” (*r* = 0.751), while the lowest between subscales ”peers” and ”physicians” (*r* = 0.509) (Table 3).

### Hospital ethical climate versus correlation with sociodemographic variables and work environments of polish nurses/midwives

The mean score for the overall hospital ethical climate was 3.62 (SD = 0.60). The highest mean score was reported in the ”peers” subscale (M = 4.02, SD = 0.59) and the lowest in the ”physicians” subscale (M = 3.27, SD = 0.74) (Table 4).

**Table 4:** Descriptive statistics of HECS-Pol and correlation with sociodemografic and work-related variables (n = 558)

Descriptive statistics			Variables																								
**Subscales**	**M**	**SD**	**Age** ^**#**^		**Gender**				**Education**					**Religious beliefs**				**Work experience** ^$^		**Satisfaction with salary** ^**±**^		**Work satisfaction** ^**±**^		**Working time**			
					**Z**		**p**			**F**		**p**			**Z**	**p**							**H**		**p**		
**Peers**	4.02	0.59	0.010	-1.075		0.282		**3.223**			**0.041**		-0.765			0.444	0.019		**0.090** ^*^		**0.228** ^***^		1.251		0.535		
**Patients**	3.83	0.62	0.074	-1.027		0.305		**3.083**			**0.047**		-0.155			0.877	**0.090** ^*^		0.041		**0.198** ^***^		**7.477**		**0.024**		
**Managers**	3.59	0.89	0.010	-0.986		0.324		**3.272**			**0.039**		-0.102			0.918	0.006		**0.120** ^**^		**0.215** ^***^		**8.775**		**0.012**		
**Hospital**	3.41	0.78	0.007	-0.703		0.482		0.269			0.764		-1.753			0.080	0.016		**0.150** ^***^		**0.211** ^***^		**11.347**		**0.003**		
**Physicians**	3.27	0.74	-0.012	-1.216		0.224		0.406			0.666		**-2.615**			**0.009**	-0.002		**0.209** ^***^		**0.240** ^***^		**13.564**		**0.001**		
**Global**	3.62	0.60	0.019	-0.608		0.543		1.894			0.151		-1.357			0.175	0.031		**0.153** ^***^		**0.263** ^***^		**12.235**		**0.002**		

M – Mean, SD – Standard Deviation, ^#^ Pearson’s r; Z – Mann-Whitney U test, F– ANOVA, ^$^Spearman’s rho; t – t-test, ^±^definitely yes – 5; definitely no – 1; H – Kruskal–Wallis H; * <0.05; ** <0.01; ***<0.001

The study results showed a positive correlation between the overall hospital ethical climate (rho = 0.153; p < 0.001), the four HECS-Pol subscales (Table 4), and satisfaction with salary. Job satisfaction was positively correlated with the overall hospital ethical climate (rho = 0.263; p < 0.001) and all subscales. Additionally, work experience was correlated with the HECS-Pol in the “patients” subscale (rho = 0.090; p < 0.05).

Education showed correlation with the level of ethical climate in the “peers” (F = 3.223; p < 0.05), “patients” (F = 3.083; p < 0.05), and “managers” (F = 3.272; p < 0.05) subscales. Also, we observed correlation between working time and the overall level of ethical climate (H = 12.235; p < 0.01) and the four HECS-Pol subscales. Furthermore, our study revealed correlation of religious beliefs on the ”physicians” (Z = -2.615; p < 0.01) HECS subscale (Table 4).

A correlation was observed between selected problems occurring in nurses’ and midwives’ work. These included, for example, lack of time for direct face-to-face care (rho = -0.170; p < 0.001), moral dilemmas in relation to patient care (rho = -0.142; p < 0.01), low salary (rho = -0.170; p < 0.01), physical (rho = -0.091; p < 0.05) and mental burden (rho = -0.099; p < 0.01). The strongest correlation was found in relations with hospital managers (rho = -0.400; p < 0.001). The higher a given problem was rated, the lower the hospital ethical climate (Table 5). In general, the correlations had low scores (0.2–0.4).

**Table 5:** The relationship between the ethical climate and nurses/midwives problems occurring in their work

Main problems occurring in nurses’ and midwives’ work ^#^		HECS-Pol	
Patients’ conditions	**-0.084** ^*^		
Patients’ expectations	-0.066		
Patients’ families expectations	-0.019		
Lack of time for direct face-to face-care	**-0.170** ^***^		
Lack of equipment and resources for high quality of healthcare	**-0.216** ^***^		
Relationships in the nursing team	**-0.397** ^***^		
Relationships in the therapeutic team, with other professionals	**-0.424** ^***^		
Relations with hospital managers	**-0.400** ^***^		
Limitations in own professional competences	**-0.182** ^***^		
Moral dilemmas in relation to patient care	**-0.142** ^**^		
Low salary	**-0.170** ^**^		
Low prestige of nurse/midwives work	**-0.220** ^***^		
Physical load	**-0.091** ^*^		
Mental load	**-0.099** ^*^		
Medical/nursing documentation requirements	-0.058		
Risk of making a mistake	-0.080		
Low professional competences of others medical professionals	**-0.217** ^**^		

^**#**^definitely yes – 5; definitely no -1; Spearman’s rho; * <0.05; ** <0.01; ***<0.001

## Discussion

In Polish settings, according to literature review, no research tool is available to assess the hospital ethical climate. The present study was conducted to examine selected psychometric properties of the Polish version of the Hospital Ethical Climate Survey, assess the hospital ethical climate as perceived by nurses and midwives working in Polish hospitals, and analyse correlations between this climate and different variables. The process of adaptation, validation and examination of the psychometric properties of the HECS-Pol provides the opportunity to fill in the gaps in the current body of knowledge about this area, opens the way for continuous, more precise and nation-wide research, and makes it possible to unify international debate on the topic as well as to align findings on the hospital ethical climate with potential improvement measures.

The two fundamental elements in the evaluation process of a measurement instrument are its reliability and validity [30]. The results obtained in this study confirm that the Polish 21-item version (with items Q1, Q2, Q4, Q8, Q9 removed from the study) of the Hospital Ethical Climate Survey is a reliable and stable tool with acceptable psychometric properties. This is in addition to completely standardised loadings on the final factor structure across the entire HECS-Pol, which demonstrate a solid structure of the tool. For comparison, in a validation study by Khalesi et al. [26], factor loadings for all hospital ethical climate items ranged between 0.50 and 0.80.

Five questions that were included in the original version of the scale (Q1: My peers listen to my concerns about patient care; Q2: Patients know what to expect from their care; Q4: Hospital policies help me with difficult patient care issues/problems; Q8: A clear sense of the hospital’s mission is shared with nurses; Q9: Physicians ask nurses for their opinions about treatment decisions) had to be removed from the HECS-Pol, because they did not meet the psychometric criteria. This may be due to cultural differences, the work organisation system of nurses and midwives or the composition of the study group.

The overall internal consistency of the Polish version of the Hospital Ethical Climate Survey in terms of Cronbach’s alpha was high (0.93) and revealed good properties of the scale. Acceptable values of Cronbach’s alpha ranged from 0.70 to 0.95 (depending on the literature), yet the values preferred for the psychometric quality of the scales ranged from 0.80 to 0.95 [30–33]. Cronbach’s alphas in different validation studies of HECS varied between 0.86 in the Greek version [27] to 0.94 in the Persian version [26].

### Hospital ethical climate as perceived by polish nurses and midwives

In our study, the overall hospital ethical climate was perceived by nurses and midwives as average (M = 3.62, SD = 0.60). Slightly better results were obtained in Finland in settings involving care of older adults (M = 3.85; SD = 0.56) [34], in Iran (M = 3.79, SD = 0.56) [35], and in the United States among nurses and social workers (M = 3.70; SD = 0.55) [36]; worse results were returned in Cyprus among registered cancer nurses (M = 3.53; SD = 0.61) [37]. This suggests that, with respect to the hospital ethical climate as perceived by nurses and midwives, differences between countries may be determined by both working and cultural conditions, for example: nursing shortage, type of hospital word, systems of values, communication system in a nursing groups and organisation, leadership style.

In the present study, the highest mean score for the hospital ethical climate was found in the ”peers” subscale and the lowest in the ”physicians” subscale. This indicates that the surveyed nurses and midwives shared good relations mainly within their respective professional groups, rather than with physicians. Effective cooperation between nurses and midwives as well as mutual understanding and readiness to assist each other in difficult situations all contribute to a more positive hospital ethical climate. In contrast, problems with communication, lack of support, issues concerning respect for other people’s opinions, mutual trust, and involvement in the decision-making process while working with physicians worsened the perceived hospital ethical climate among nurses and midwives and the quality of health care [37]. Similar results were reported by Suhonen et al. [34] and Teraz et al. [35]. Our findings are similar to those in the study by Bartholdson et al. [38], where nurses felt that they did not have influence on medical decisions. What is more, health care professionals needed teamwork, respect, good communication, and reflection to effectively deal with ethical issues [38]. Considering the fact that in our study the subscale “physicians” received the lowest score, cooperation with physicians is an area which demonstrates considerable room for improvement. Physicians and nurses do not cooperate and provide care separately from each other – this can affect the quality of their services as well as the hospital’s ethical climate [35].

Poikkeus et al. [36] noticed a need to support the ethical competencies of nurses. This is becoming highly important as our research has shown how important it is to support nursing staff in difficult ethical decisions. Furthermore, managers should develop organisational recommendations and policies, e.g. case studies or descriptions of ethical competencies, in order to promote debate on ethical issues and to support ethical competencies of midwives and nurses in their work with patients [36], this could help to create a more positive ethical climate in the hospital. Managers should reduce the impact of ethical dilemmas on nurses’ work by providing social support, developing and training managers’ ability to understand nurses’ and midwives’ needs, and learning how to communicate and solve said ethical dilemmas [39]. The perception of the hospital ethical climate may be impacted by managers’ ethical competencies and their involvement in support to solve everyday ethical problems related to patient care. Literature review showed the importance of teamwork, workload balancing, and staff relations for a workplace culture which offers room for the development of person-centred relationships [40].

A positive correlation was found between respondents’ work experience and the subscale “patients”. This may be related to the multitude of ethical problems occurring in nurses’ and midwives’ work, where, with increasing seniority, they shape and develop their individual techniques of communicating with patients, making decisions or solving ethical problems.

Younger and less experienced nurses sustained higher levels of stress and more frequently reported and struggled with ethical issues in their work [12]. Notwithstanding, no statistical difference was found between overall values of the hospital ethical climate perceived by nurses and midwives and their age, occupational status, religious beliefs, place of residence, gender, and education. Demographic variables such as gender, age, and education were not significantly correlated with the mean score of ethical climate in the study by Ghorbani et al. [22], just as age and marital status in the study by Karca et al. [17].

In our study, education correlated with the hospital ethical climate in subscales “peers”, “patients” and “managers”. A better perception of this climate was reported by participants with the Master’s degree or other educational background (doctoral degree, vocational education) as compared to those with the Bachelor’s degree. Similar results were obtained by Ghorbani et al. [22], where mean scores of ethical climate increased as the educational level grew. In contrast to our study, Constantina et al. [19] found that nurses with higher education reported poorer average perception of the hospital ethical climate across all dimensions. A certain educational level (resulting in broader knowledge and skills, or work in an interdisciplinary team) entailed higher expectations and needs in the unit where nurses and midwives worked. Also, the level of education and work experience significantly influenced the perception of the ethical climate in the work environment [41]. In our study, nurses and midwives with the MA degree, during their university education had acquired knowledge and skills necessary to plan and organise nursing/midwifery work, which means that they can effectively contribute to the ethical climate in their work environment.

According to scientific evidence, job satisfaction is shaped by salary, working conditions, and personal development, and is related to ethical climate [9, 17]. Higher work satisfaction contributes to a more positive hospital ethical climate. This is in line with the present study, as the hospital ethical climate was correlated with work and salary satisfaction, and also with working time. As indicated by Abou Hashish [16], a positive ethical climate promotes nurses’ and midwives’ sense of determination and reduces the risk of occupational burnout, thereby improving the organisation of nursing work. What is more, with adequate support facilitated by ethical climate, nurses are more likely to provide high-quality care to patients, which in turn enhances their satisfaction with work, fewer errors occurring in nursing practice [42]. There is a relationship between the hospital ethical climate and self-perceived competences, the intention to resign from work, and satisfaction with the performed tasks in terms of the quality of health care [7].

In the present study, correlation was found between the hospital ethical climate and problems in nurses and midwives’ work for example: lack of time for direct face-to-face care, moral dilemmas in relation to patient care, low salary, physical and mental burden. In this context, the level of hospital ethical climate is not a permanent feature. Rather, it is affected by all of the indicated aspects, in particular the involvement of hospital managers, their leadership skills and team-leading methods, relations with other health care professionals, and workplace culture – this is in line with other published studies [34, 42, 43]. This result suggests possible directions for improving the organisation of the health care system and reveals the need to create an ethical hospital climate with all its important elements as included in the Hospital Ethical Climate Survey (HECS).

### Limitations

There are several limitations to our study. First of all, they may result from the fact that the studied group represented only two (the eastern and southern) regions of Poland. Second, some of the surveys were collected early into the COVID-19 pandemic, which may have impacted on how the hospital ethical climate was perceived by nurses and midwives. Furthermore, our study was cross-sectional, which made it difficult to determine the cause and effect. An in-depth analysis is therefore needed, e.g. utilising a mixed-method approach where quantitative analysis is supplemented with qualitative one.

## Conclusion

The current research, conducted for the first time in Poland to assess the hospital ethical climate, has shown that the 21-item Polish version of the Hospital Ethical Climate Survey (HECS-Pol) is a reliable tool well adapted to cultural conditions and characterised by acceptable psychometric properties.

The study results, based on responses from Polish nurses and midwives, indicated a moderate level of hospital ethical climate. The aspect that should be specifically addressed in this regard are nurses’/midwives’ relations with physicians and hospital managers. At the same time, the relationships shared by nurses and midwives were generally positive, which might be regarded as a factor potentially contributing to an improved hospital ethical climate.

Our study results echo the findings of studies conducted in other countries: the hospital ethical climate correlates with job and salary satisfaction and with working time. Moreover, our study has indicated the need to monitor a number of work-related problems experienced by Polish nurses and midwives as well as to investigate how these problems can be tackled through the improvement of the hospital ethical climate. As far as remuneration of nurses and midwives is concerned, new criteria need to be developed, taking into account education, experience, and many other factors, in order to ensure fair payment for their work. This topic is now being broadly discussed by different stakeholders in Poland. It is also necessary to take care of the future generations of nurses and midwives by increasing higher education quotas and using incentives in the form of scholarships to make up for staff shortages in many hospitals. And finally, nursing and midwifery students and managers should be trained on how to build a positvie ethical climate in the workplace.

### Implications for nursing management

This study may contribute to the dissemination of the Polish version of the Hospital Ethical Climate Survey (HECS-Pol) in Poland and encourage researchers to use it to assess the ethical climate of hospitals, analyse its correlation with variables such as demographic factors, occupational burnout, moral sensitivity, moral stress, and job satisfaction, and diagnose areas in the hospital which require organisational changes as well as to evaluate their effectiveness. By using the Polish version of the Hospital Ethical Climate Survey, hospital managers can create strategies to improve ethical climate, provide education opportunities, train their employees in adjusting their needs and, in consequence, improve the overall quality of nursing care and job satisfaction among nurses and midwives and in their work environment.

## Electronic supplementary material

Below is the link to the electronic supplementary material.

Supplementary Material 1

## Data Availability

All the data generated and analysed as part of this study are not publicly available to protect participants’ privacy and confidentiality; they can be obtained from the corresponding author at a reasonable request.

## Abbreviations

HECS: Hospital Ethical Climate Survey
HECS-Pol: Polish version of the Hospital Ethical Climate Survey
STROBE: The STrengthening the Reporting of OBservational studies in Epidemiology
KMO: Kaiser-Meyer-Olkein
CFA: Confirmative Factor Analysis
RMSEA: Root Mean Square Error of Approximation
SRMR: Standardised Root Mean Square Residual
CFI: Comparative Fit Index
TLI: Tucker-Lewis Index
IFI: Bollen’s Incremental Fit Index
