# Supplementary figures and images for: Hospital ethical climate survey - selected psychometric properties of the scale and results among polish nurses and midwives
Source: BMC Nurs. 2022 Nov 2;21:295. doi: 10.1186/s12912-022-01067-x (PMC9628138; doi:10.1186/s12912-022-01067-x)

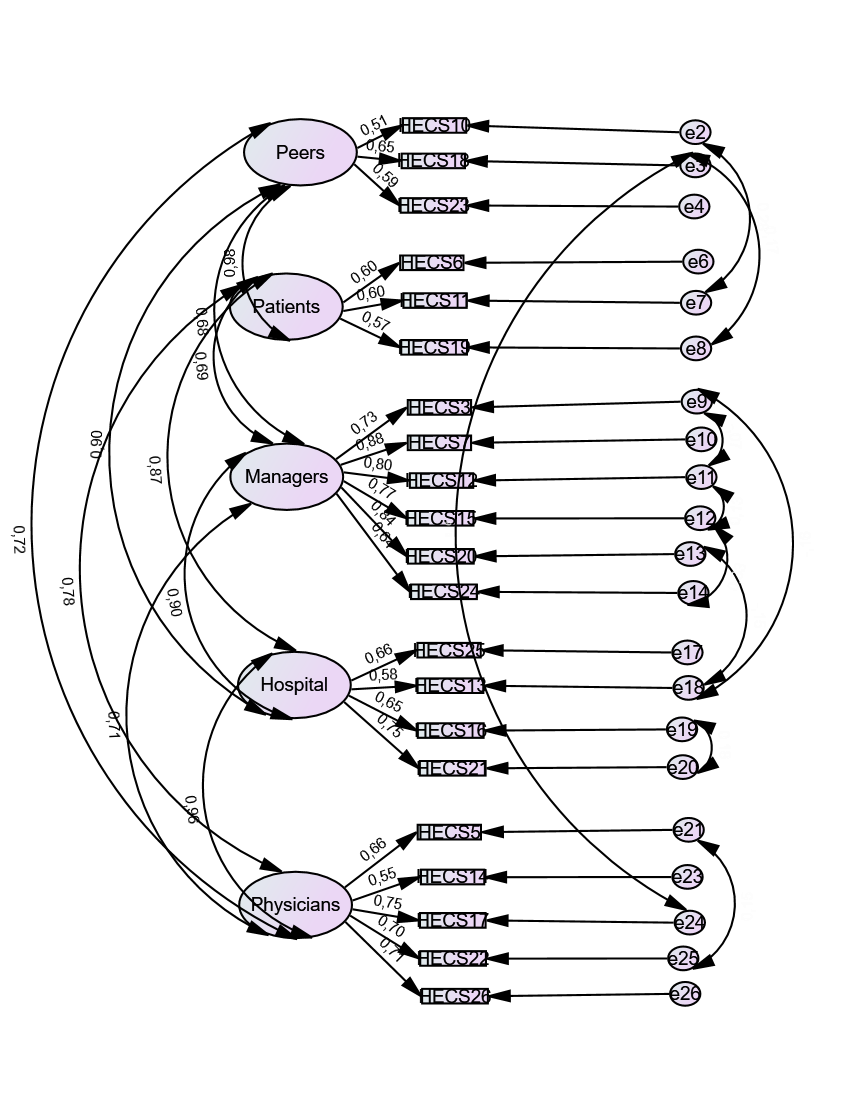


Additional file 2. Confirmatory factor analysis of the HECS-Pol

Supplement: Supplementary file 1 — Supplementary Material 1 [file 12912_2022_1067_MOESM1_ESM.docx]
